# Enhancing Caregivers’ Quality of Life Through a Web-Based Person-Centered Solution (TechQoL4Carers): Protocol for a Mixed Methods Pilot Trial

**DOI:** 10.2196/86602

**Published:** 2026-02-05

**Authors:** Betania Groba, Patricia Concheiro-Moscoso, María del Carmen Miranda-Duro, Manuel Lagos-Rodríguez, Javier Pereira, Laura Nieto-Riveiro

**Affiliations:** 1 CITIC. Research group TALIONIS, Faculty of Health Sciences, Universidade da Coruña, As Xubias, s/n, A Coruña, 15071, Spain, 34 981167000 ext 5870

**Keywords:** quality of life, informal caregivers, web-based platform, wearable devices, activities of daily living, person-centered care

## Abstract

**Background:**

Informal care is a social challenge that impacts the daily life and quality of life (QoL) of caregivers. While care has evidence of positive aspects, it can also have negative impacts on mental, physical, economic, and social well-being. Nowadays, health, social, and care systems for informal caregivers are needed, from a person-centered perspective, to promote their QoL, health, and empowerment. Technology is a promising tool to provide personalized services.

**Objective:**

This study aims to develop an innovative care-centered technology solution to enhance caregivers’ QoL, care impact, occupational balance, health self-management, and empowerment.

**Methods:**

A mixed methods pilot trial was designed as a single-arm open-label study. Participants will be caregivers of people with disabilities or older people, recruited through direct care centers. Caregivers will engage in a participatory process of developing, testing, and validating the TechQoL4Carers project platform, *CuidaconTIC*; a web-based platform to improve their QoL, impact of care, occupational balance, health self-management, and empowerment. The development of this trial will refine the *CuidaconTIC* platform, which is based on person-centered development, participatory design techniques, and an iterative process. A total of 54 caregivers will participate in a 3-month intervention involving the use of the *CuidaconTIC* platform and the Xiaomi Smart Band 7 wristband. Standardized assessment tools (EQ-5D-5L, Care-Related Quality of Life, Zarit Burden Interview, Caregiver Strain Index, Occupational Balance Questionnaire-Spanish Version, Psychological Empowerment Instrument, and the System Usability Scale) and a self-designed tool (Satisfaction-Q) will be used at 3 time points to collect information about usability, satisfaction, and project variables. Continuous information will be obtained from the platform (My week-Q) and the wearable wristband (physical activity and sleep). An interview will be conducted to gain in-depth knowledge about participants’ perspectives. This study was approved by the Ethics Committee for Research and Teaching of the Universidade da Coruña (2023_019) and registered on ClinicalTrials.gov. Participation will be entirely voluntary, with informed consent obtained from each participant. Detailed information sheets and informed consent forms will be provided. The data of the participants will be collected in a pseudonymized form. Once the study has been completed, any possibility of participant identification will be eliminated.

**Results:**

Financial support for this project was received on December 1, 2022. This protocol was submitted after data collection but before analysis. Data collection began in May 2024 and ended in March 2025. By October 2025, 62 participants had been recruited. We expect to publish our results in June 2026.

**Conclusions:**

This protocol focuses on the study of the social challenge of quality of life of caregivers, mostly women, and on reducing the digital gap and promoting inclusion, through a service that places people and care at the center.

## Introduction

### Background and Rationale

Care is considered an occupation in daily life, encompassing a wide range of responsibilities linked to support in activities of daily living and/or instrumental activities of daily living [1,2]. In this regard, informal care, often provided by family members, plays a crucial role in providing support to individuals with long-lasting needs (chronic illnesses, disability, age-related dependency, among others), and in many cases, they must face decision-making together with health and social services [3,4].

In recent years, the combination of social, demographic, and economic factors has increased informal care across Europe [5]. Statistics indicate that approximately 14.4% of the European population aged 18 to 74 are informal caregivers, providing up to 80% of all long-term care for dependent individuals [6]. In particular, Spain is among the European countries with the highest prevalence of informal caregivers, with 15.3% of the population engaged in this role and 52.9% of informal caregivers dedicating more than 20 hours per week to care [7,8]. Furthermore, literature states that the most common profile of an informal caregiver is a woman (n=32,590, 63.28%) aged between 45 and 64 years (n=18,814, 55%) [6,9].

Although care can be a source of great personal satisfaction, it also poses numerous challenges [10]. Caregiving tasks can significantly impact the quality of life (QoL), well-being, and health of informal caregivers. The burden of long-term care may lead to physical and cognitive difficulties, as well as mental health issues [10,11].

Evidence suggests that women, older caregivers, and spouses are likely to experience high levels of stress related to their care roles [9,12]. Factors such as task overload, insufficient support, financial difficulties, restrictions in daily activities, and social exclusion can lead to chronic stress [13,14]. This prolonged situation, when combined with high levels of emotional exhaustion, sleep disturbances, and physical issues, can lead to “caregiver’s burnout syndrome or caregivers’ burden” phenomenon [13,15]. Additionally, mental health issues are approximately 20% more prevalent among informal caregivers than noncaregivers, especially among those who provide intensive care (more than 20 h/wk) [5,16].

Informal care is also related to occupational balance, habits, and routines of the caregiver. Providing informal care directly affects the caregiver’s daily life [17]. Some authors mention that care responsibilities can be a barrier to accessing education and paid employment [17,18]. In fact, many caregivers, due to difficulties in reconciling work and care duties, may be forced to reduce their working hours and/or leave paid employment, leading to financial, social, and personal difficulties [10,19]. Specifically, statistical data suggest that women aged between 35 and 44 are most affected by this situation, finding themselves obliged to reconcile with part-time employment (60.2%) or to stop working (31.4%) [8].

Furthermore, caregivers also face challenges in dedicating time to other meaningful occupations and maintaining their healthy lifestyle habits [2,17]. Specifically, statistical data shows that Spanish caregivers have reduced their leisure activities by 77%, their time for social participation by 47.88%, and their time for personal self-care by 60.34% [8].

In this way, the repercussions of care can make informal caregivers susceptible to health issues that may lead to chronic diseases [11]. Furthermore, the increase in the number of older informal caregivers also highlights the need for social and health care support for them [5]. These circumstances, along with the implementation of person-centered care (PCC) principles, underscore the importance of decision-makers, policymakers, and professionals recognizing, respecting, and supporting informal caregivers by seeking and developing cost-effective, sustainable, and preventive solutions [20,21].

Various studies have focused on the development of intervention programs aimed at reducing care burden, providing social support, or improving caring skills [1,14,19]. However, despite their potential, these programs have some limitations related to difficult access, lack of information, or insufficient support for caregivers to participate effectively [16]. Consequently, there is a need for innovative methods that eliminate these barriers [22]. Thus, Information and Communication Technologies offer the possibility to provide support and services to caregivers in real time [22].

In recent years, the development and evaluation of technological solutions aimed at the informal caregiving population has become an area of growing interest [23]. The literature highlights that tools such as mobile applications, online educational programs on caregiving, health monitoring devices, task management systems, and emotional support networks are accepted by informal caregivers as they facilitate the management of care, improve the quality of care, reduce emotional and physical burden, and enhance their well-being [20,24].

In this regard, Tan et al [25], in their scoping review, report that the use of technological tools by informal caregivers contributes to a reduction in caregiving burden and a slight improvement in the quality of life of caregivers. While various studies emphasize the benefits of digital solutions to improve adherence to treatments for dependent individuals, optimize care task management, and highlight the importance of home assistive technology [26].

Despite advancements in the implementation of technological solutions, significant barriers to their adoption remain, particularly among older caregivers [25]. Studies such as those conducted by Egan et al [27] and Xiong et al [28] point out certain concerns of informal caregivers. These include fears that these technologies may replace human contact, a lack of familiarity with these technologies, the costs associated with some applications or technological services, the lack of technological adaptation, sustainability, the rapid evolution of technology, as well as the design and usability of these systems.

Furthermore, many mobile applications do not offer personalized content, leading caregivers to perceive the information as irrelevant or overly complex for their needs [29]. As noted by Premanandan et al [30], current technological solutions primarily focus on managing the care of dependent individuals but fail to address the specific needs of the caregivers themselves. This trend is particularly evident in studies centered on caregivers of individuals with dementia, where Information and Communication Technologies solutions have been designed without a comprehensive approach that also considers the emotional and well-being needs of informal caregivers [31]. Additionally, some applications aimed primarily at caregivers, such as the CareFit app, which encourages caregivers to engage in regular physical activity at home, have limitations, including the lack of time caregivers have to carry out activities, difficulties in understanding how to perform the activities, and the feeling of insecurity when carrying out those activities at home [32].

Thus, the literature emphasizes the importance of involving informal caregivers in the design and development process of technological solutions to ensure that these meet their needs effectively, are accessible, easy to use, and sustainable in the long term [22,28]. Person-centered design is crucial for improving the health, maintaining the quality of life, and strengthening the well-being of caregivers, which in turn positively impacts the care provided, at health, social, and economic levels [33].

In this regard, this project aims to address these challenges by creating a technological solution that improves the quality of life and health status of informal caregivers, as well as being aware of the implications and repercussions that continuous care has on informal caregivers.

### Aims, Objectives, and Hypotheses

According to the existing literature and the main conclusions of the background, there is a clear need to develop a technological solution specifically designed for informal caregivers. The hypothesis is that this innovative technological solution will have a positive impact on their QoL, impact of care, occupational balance, self-management of health, and empowerment.

The primary objective of this pilot trial is to develop and evaluate an innovative technological solution aimed at enhancing the QoL, impact of care, occupational balance, self-management of health, and empowerment of informal caregivers of older people and/or people with disabilities. The main question is: Will the routine use of the TechQoL4Carers platform have a positive impact on the daily life of informal caregivers?

The specific objectives of this project are (1) to develop an innovative technological solution for informal caregivers aimed to improve their QoL, impact of care, occupational balance, self-management of health, and empowerment; (2) to assess the impact of the platform on the QoL, care, self-management of health, and empowerment of informal caregivers; (3) to test and evaluate the contents, usability, quality, and satisfaction with the platform by informal caregivers; and (4) to identify potential barriers and necessary changes as well as facilitators to use the platform.

## Methods

### Study Design

A mixed methods pilot trial was designed as a single-arm open-label study. Informal caregivers will participate in a participatory process of developing, testing, and validating a technological platform to improve their QoL, impact of care, occupational balance, self-management of health, and empowerment.

The 3-month intervention will be based on the use of the TechQoL4Carers technology platform, called *CuidaconTIC* [34], and the Xiaomi Smart Band 7 wearable wristband. Formal assessments using standardized tools will be conducted as follows: baseline (preintervention: t_0_), mid-intervention (at 6 wk: t_2_), and postintervention (t_4_).

Throughout the intervention (t_1_ to t_3_), continuous information will be obtained through data from the platform and the Xiaomi wearable wristband. A qualitative evaluation will be conducted to gain in-depth knowledge about participants’ perspectives on the intervention and study procedures (t_4_).

The trial has been registered on ClinicalTrials.gov with the identification number NCT06226285 [35]. As this is research with people, in this case, caregivers, a favorable report has been previously obtained from the Ethics Committee for Research and Teaching of the Universidade da Coruña (code: 2023_019).

In addition, the SPIRIT (Standard Protocol Items: Recommendations for Interventional Trials) statement [36] and the CONSORT-EHEALTH (Consolidated Standards of Reporting Trials of Electronic and Mobile Health Applications and Online Telehealth) guidelines [37] were adopted for the protocol design. A summary of the trial, based on the World Health Organization (WHO) Trial Registration Data Set [38], can be found in Multimedia Appendix 1.

### Participants, Recruitment, and Eligibility Criteria

The participants in this study will be caregivers of people with disabilities or older people. The sample will be recruited through direct care centers for people with disabilities and older people.

Depending on the functioning of the entities, a meeting will be held in the centers, individually or in groups, with the persons preselected by the centers and the research team to provide detailed information on the project and possible participation. In this meeting, the inclusion and exclusion criteria will be explained and a paper information sheet will be distributed.

Individuals may express their interest in participating through the e-mails included in the information sheet or through the reference persons in each entity. Subsequently, an individual meeting will be held with the candidates to clarify any doubts and, if applicable, to confirm participation in the project by signing the informed consent form.

Informal caregivers will be included in the study if they are 18 years of age or older; perform the role of informal caregiver, are the main caregiver; are living with the person receiving the care; regularly use a smartphone; and commit themselves to wearing an activity wristband regularly during the project. The exclusion criteria were those who had been an informal caregiver for less than 1 year, were the main caregiver of more than 1 person in a situation of dependency, or had modified legal capacity.

**Figure 1.**
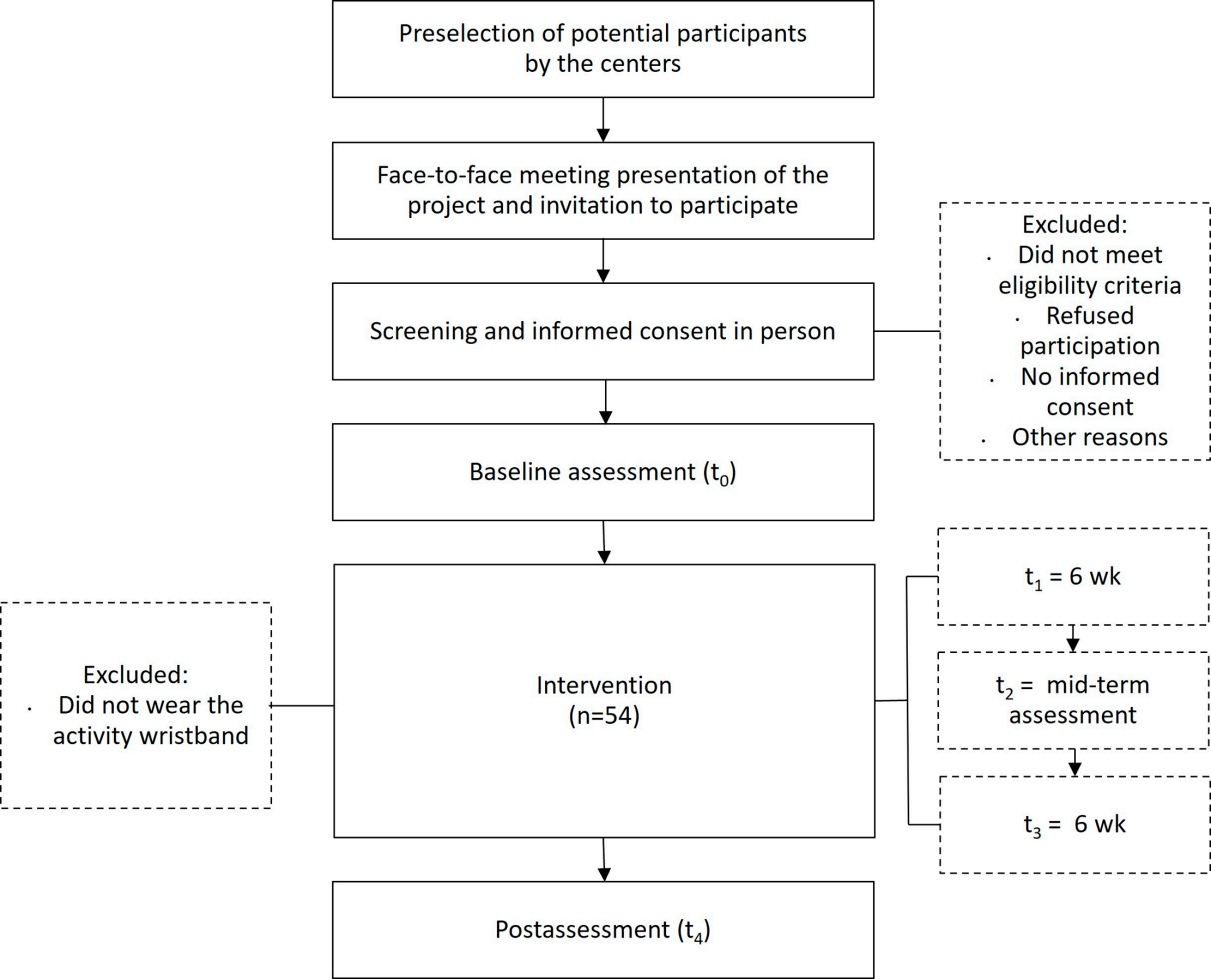
Study flowchart.

Figure 1 shows visually the study flowchart described above.

### Sample Size

To determine the necessary sample size for this study, the G*Power software was used. Based on similar studies and assuming that caregivers’ QoL is the primary outcome of this study (with all other variables treated as secondary and/or exploratory outcomes), power analysis was performed [39]. An a priori power analysis was conducted using a paired t test on the primary outcome, as an improvement in informal caregivers’ QoL was expected after the intervention. A moderate effect size (Cohen *d*=0.5), a significance level of .05, and a statistical power of 80% (1−*β*=.80) were assumed. Under these assumptions, a sample of 54 caregivers who complete the study (*df*=53) provides power greater than 80% to detect the expected effect.

Given the multiplicity of the planned analyses, confirmatory inference will be restricted to the primary outcome. Secondary and exploratory outcomes will be interpreted with caution, without applying a formal adjustment for multiple comparisons, and emphasis will be placed on effect sizes and their 95% CIs.

In addition, an attrition rate of up to 20% is anticipated (due to dropouts, absence from the postintervention assessment, or technical problems with the wearable device or platform). Therefore, we plan to recruit approximately 65 informal caregivers, so that at least 54 are expected to complete the study and be included in the primary analysis.

### Ethical Considerations

The project complies with the fundamental principles for research with human beings established in the Declaration of Helsinki, the Oviedo Bioethics Convention, and the Universal Declaration on the Human Genome and Human Rights.

The performance of this study will respect the applicable regulations of ethics regarding research with humans. Also, for data protection, we will follow the Regulation (EU) 2016/679 of the European Parliament and of the Council of April 27, 2016, on the protection of natural persons regarding the processing of personal data and on the free movement of such data and the national regulations on data protection.

To guarantee these aspects, the research protocol was submitted to the Ethics Committee for Research and Teaching of the Universidade da Coruña. A favorable report from this committee was obtained (2023_019).

The group of participants in the study will be adults in an informal caregiver’s role. Participation in this project will not implicate any risk or damage to the participants, who will (1) cover baseline, mid-term, and end-line surveys, (2) wear a noninvasive wearable device for the duration of the validation, (3) interact and use the platform, and (4) participate in individual semistructured interviews.

Particular attention will be paid to ethical issues: aspects that guarantee data protection and informed and voluntary participation.

The research group is committed to guaranteeing the confidentiality and protection of the data obtained from the participants in the project. The data of the participants will be collected in a pseudonymized form to allow validation tests at different stages of the development process. The interviews will be audio-recorded, with the consent of the participants. A transcript of the interviews will then be made, and the identities of the participants will be coded. The information that might allow identification shall be anonymized. Subsequently, the audios will be deleted.

The access to the identification of the participants will be exclusively carried out by the research team. Once the study has been completed, any possibility of participant identification will be eliminated.

The platform’s cybersecurity mechanisms will be identified and implemented following current legislation.

Participation will be completely voluntary, and informed consent will be obtained from each person. For this purpose, detailed information sheets and an informed consent form will be provided to explain the participation in the study. There is no obligation to complete the experiment, so participants can withdraw from it at any time.

Individual participant data will be available for sharing with the scientific community, upon request and after the acceptance for publication of the main findings from the final data set.

The data will be completely anonymized for sharing. The dataset will include only the results of quantitative variables.

Finally, for validation testing of the platform, it will be necessary to obtain a robust prototype. This prototype will be previously tested by professionals of the team to ensure the absence of unexpected behaviors of the application that may generate discomfort to participants.

The cost of the Xiaomi Mi Band 7 wristbands will be supported by the project team, and people will have to authorize the capture of data from this device (physical activity and sleep).

Participants will not receive monetary compensation. As compensation for their time, participants will receive the Xiaomi Mi Band 7 wristband used in the study at no cost (ie, they may keep the device after participation). Compensation is not contingent on completing all study procedures; participants may withdraw at any time without penalty.

### Intervention

The intervention lasts 3 months and is based on the use of *CuidaconTIC* and the Xiaomi Smart Band 7 activity wristband. Participants will regularly use *CuidaconTIC* services and wear the Xiaomi Smart Band 7 wristband.

*CuidaconTIC* is a technological platform for caregivers developed through the TechQoL4Carers project [34]. The research team designed and developed *CuidaconTIC* through an iterative and participatory process with informal caregivers, the platform’s end users, and other actors involved in care, such as health science professionals.

The platform is integrated into both a website and a mobile application. The services offered include a summary of the individual’s health status, training content, advice, and personalized recommendations based on their QoL, care situation, occupational balance, self-management of health, and empowerment. It also allows for synchronization with the Xiaomi Smart Band 7 activity wristband. *CuidaconTIC*, 1.0 version, will be used for the pilot project. After the trial results are integrated, the final version of the platform will be obtained. The main services of the *CuidaconTIC* platform are: My daily summary, My health, My week, I take care of myself, and I take care of you.

Caregivers are expected to access and use the TechQoL4Carers platform at least once a week to complete the weekly questionnaire and consult the training content related to self-care and care provision at least once a month.

The research team will provide caregivers with the Xiaomi Smart Band 7 wristband to capture health-related data and synchronize it with the platform. Participants are expected to use the wristband daily during the 3-month intervention. These data will be combined with other information collected through subjective measurements to provide personalized content and recommendations.

### Outcomes and Measurements

Participants will complete the quantitative evaluations at 3 points in time: baseline (t_0_), intermediate (t_2_), and final (t_4_) through the *CuidaconTIC* platform in its evaluation section. For the final assessment, people may participate in a semistructured interview. The evaluations at these time points will be face-to-face at the reference centers or, if preferred, at the facilities of the research center.

The weekly evaluation will be performed through the *CuidaconTIC* platform and will be reminded through notifications in the mobile application.

The schedule of enrollment, interventions, and assessments, based on the proposal of the SPIRIT statement [36], can be consulted in Table 1.

**Table 1.** Schedule of enrollment, interventions, and assessments.

	Enrollment	Preassessment	Intervention (3 mo)			Postassessment
	–t_1_	t_0_ (baseline)	t_1_^a^	t_2_^b^	t_3_^c^	t_4 _(post intervention)^d^
Enrollment						
Informed consent	✓					
Configuration						
Wristband delivery	✓					
*CuidaconTIC* settings	✓					
Intervention						
*CuidaconTIC* platform			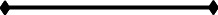			
Xiaomi Band 7			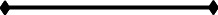			
Assessments						
EQ-5D-5L		✓		✓		✓
CarerQoL^e^		✓		✓		✓
ZBI^f^		✓		✓		✓
CBI^g^		✓		✓		✓
OBQ-E^h^		✓		✓		✓
PEI^i^		✓		✓		✓
Physical activity			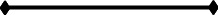			
Sleep			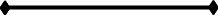			
My week-Q^j^			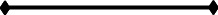			
Satisfaction-Q^k^						✓
SUS^l^						✓
Interview						✓

a t_1_: 6 weeks of intervention (1-6 wk).

b t_2_: mid-term assessment.

c t_3_: 6 weeks of intervention (7-12 wk).

d t_4_: 1-month post intervention.

e CarerQoL: Care-Related Quality of Life.

f ZBI: Zarit Burden Interview.

g CBI: Caregiver Strain Index.

h OBQ-E: Occupational Balance Questionnaire-Spanish version.

i PEI: Psychological Empowerment Instrument.

j My week-Q: *CuidaconTIC* weekly Questionnaire.

k Satisfaction-Q: *CuidaconTIC* Satisfaction Questionnaire.

l SUS: System Usability Scale.

### Characteristics of Participants

#### Sociodemographic Questionnaire

A sociodemographic questionnaire was developed with the following variables: age, gender, marital status, educational level, residential environment, household unit, employment situation, years in the role of caregiver, supports, type of support, relationship with the person receiving care, main difficulties, diagnosis, and age of the cared-for person.

#### Primary Outcome Measures

##### EQ-5D-5L

EQ-5D-5L is a self-administered scale to assess perceived health-related QoL [40-42].

Health state is obtained from the EQ-5D-5L, through its descriptive system with 5 dimensions: mobility, self-care, usual activities, pain/discomfort, and anxiety/depression. The dimensions are scored on a 5-point Likert scale reflecting the level of severity (no problem, slight problems, moderate problems, severe problems, and extreme problems or unable to perform). The health profile or health state describes the level of severity that the person perceives in each dimension and it is represented through an individual code for each dimension scored between 1 and 5 or through a single 5-digit code (ranging from 11111 to 55555).

Overall health is derived from a visual analog scale (EQ-VAS) on perceived health, from 0 to 100. In this way, the person scores his or her health on a given day, assigning it a value between 0 (the worst health you can imagine) and 100 (the best health you can imagine).

##### Care-Related Quality of Life Instrument

Care-related quality of life (CarerQoL) is a self-administered instrument to measure care-related QoL and the impact of the care on caregivers [43].

CarerQoL will be measured through 5 negative and 2 positive dimensions (CarerQoL-7D). The 7 dimensions are fulfillment from caregiving, relational problems, mental health problems, problems with combining daily activities with care, financial issues, perceived support, and physical health problems [44]. They are scored on a Likert scale with 3 values indicating the severity level (no, some, and a lot). For negative dimensions, the values are 0 (a lot), 1 (some), and 2 (no); while for positive dimensions, the values are reversed 0 (no), 1 (some), and 2 (a lot). Score range: 0 to 14. A tariff for the CarerQoL based on Dutch preferences is also available which allows a weighted sum score, ranging from 0 to 100 [44,45]. The higher the score on the dimensions, the better the situation of care.

The CarerQoL-VAS allows rating how happy the person feels at that moment as a measure of general well-being. This construct is scored on a horizontal VAS range from 0 (completely unhappy) to 10 (completely happy). The higher the score, the better the situation in terms of well-being.

### Secondary Outcome Measures

#### Zarit Burden Interview

Zarit Burden Interview is a questionnaire designed for the caregiver population to assess the level of burden and detect possible caregiving stressors [46-49]. The 22-item version is used, scored from 1 to 5.

The score range is 22 to 110. Score interpretation is as follows, with 2 cut-off points: no burden, 22 to 46 points; mild burden, 47 to 55 points; and severe burden, 56 to 110 points.

#### Caregiver Strain Index

The Caregiver Strain Index or Robinson Index is a 13-item tool for caregivers to measure perceived strain, and detect burden, and possible stressors related to care [50-52]. Each item is answered in a dichotomous way: yes or no.

The score range is 0 to 13. Scores of 7 or more identify caregivers at risk for high caregiving strain or burden.

#### Occupational Balance Questionnaire, Spanish Version

Occupational balance will be measured through Occupational Balance Questionnaire-Spanish version [53,54]. This self-administered tool of 13 items is scored on a 6-step ordinal scale, ranging from 0 (completely disagree) to 5 (completely agree). It evaluates the person’s current experience with occupational balance, considering the quantity, variability, and meaning attributed to activities of daily living.

The score range is 0 to 65. The results show that the higher the score, the higher the occupational balance.

#### Psychological Empowerment Instrument

The Psychological Empowerment Instrument adapted will be used to assess changes in informal caregivers’ perceptions of empowerment.

It is a self-administered instrument that measures psychological empowerment in the work environment, considering 4 cognitive dimensions: competence, meaning, self-determination, and impact [55,56]. The Spanish version of the Psychological Empowerment Instrument comprises 13 items divided among the 4 dimensions and scored on a 7-point Likert scale [57]. This version will be used in the study replacing the references to work and the role of the workers with terms associated with care and caregivers.

The score range is 7 to 91 points. The higher the score, the higher the level of psychological empowerment.

#### Activity Wristband: Xiaomi Smart Band

In the study, participants will use the Xiaomi Smart Band [58] activity wristband regularly during the 3 months of the intervention.

Version 7 of the Xiaomi Smart Band activity wristband allows the recording and monitoring of health-related variables: physical activity, sleep, heart rate, blood oxygen saturation (SpO₂), women’s health, and stress [58].

The wristband will be automatically synchronized with the TechQoL4Carers platform. The variables that will be monitored during the project are: physical activity (number of steps, distance in meters, and activity in min) and sleep (light sleep, deep sleep, rapid eye movement sleep, and time awake after sleep onset in min).

#### My Week Questionnaire

My Week Questionnaire is a questionnaire designed by the research team included on *CuidaconTIC*. Participants could self-administer it weekly to identify changes perceived in daily functioning based on the variables studied: QoL, impact of the care, occupational balance, self-management of health, and empowerment.

The development of this questionnaire has been based on previous surveys developed by the research team [59], but it has been adapted to caregivers for this study.

My Week Questionnaire is integrated with 9 items that are answered using a 6-point Likert scale, ranging from 0 (completely disagree) to 5 (completely agree). The score range is 0 to 45 points. The higher the score, the greater the perception of positive changes in QoL-related factors.

#### *CuidaconTIC* Satisfaction Questionnaire

The *CuidaconTIC* Satisfaction Questionnaire will be administered at the end of the intervention to assess end-user satisfaction with the project and the technology. The questionnaire has been adapted for this study based on the Technology Satisfaction Factor Loadings questionnaire [60].

It is a self-administered questionnaire consisting of 16 items grouped into 4 factors (competence, assessment, performance, and TechQoL4Carers platform), rated on a 6-point scale, ranging from 0 (completely disagree) to 5 (completely agree). The score range is 0 to 80. The higher the agreement with the statements, the higher the satisfaction with the project.

#### System Usability Scale

The System Usability Scale is a tool used to measure subjective perceptions of technology usability [61]. It contains 10 items scored on a 5-point Likert scale, from 0 (strongly disagree) to 4 (strongly agree). The items are statements about the effectiveness, efficiency, and satisfaction with a system or product.

The score range is 0‐100. The higher the score, the higher the perceived level of usability of a product or system. Scores of 70 or more identify acceptable products [62] with a good (70-85) and excellent (85 or more) level of usability.

#### Semistructured Interview

At the end of the project, a semistructured individual interview will be conducted with each caregiver. The interview guide has been designed to elicit the meaning that the caregivers give to the caregiving experience, to the participation in the project, to the use of technology in the project, and the impact of this project on their daily life (QoL, impact of care, occupational balance, self-management of health, and empowerment).

This interview will take place in the collaborating entities, in a quiet and comfortable place, and at a time that suits the needs of the participants. It will be recorded with a digital device to facilitate the literal transcription. This information will be coded and at the end of the study, the transcriptions will be anonymized and the audio will be deleted.

The interview guide can be found in Appendix B in Multimedia Appendix 2.

### Analysis

The statistical analysis of the data will be performed using R software (version 4.4.3 [63]). All data will be cleaned and processed prior to analysis, and a range of statistical procedures will be performed based on the study hypotheses and objectives. The significance level for hypothesis testing is set at 5%. Numerical variables will be presented as mean and SD, along with range, minimum, and maximum values, while categorical variables will be presented as absolute frequency and valid percentage.

After performing a descriptive analysis of the variables, we will determine the distribution of the data using formal tests of normality: the Kolmogorov-Smirnov or Shapiro-Wilk tests, as appropriate. This will help decide whether parametric or nonparametric statistical methods are used. Parametric tests, such as the paired *t* test, ANOVA, and Pearson correlation, will be used to analyze data if normality is confirmed. Otherwise, nonparametric tests, including the Wilcoxon signed-rank test, Friedman and Spearman rank correlation, will be employed. Pre- and postintervention changes in the primary outcome and in the other continuous variables will be analyzed using a paired Student *t* test or, where appropriate, the Wilcoxon signed-rank test. The effect size of within-subject changes will be estimated using Cohen *d*, in order to quantify the magnitude of the observed changes. In addition, 95% CIs for these effect sizes will be reported. It is important to note that QoL, considered the primary outcome of the study, will be used for formal confirmatory inference to control the type I error rate; analyses of other outcomes will be considered secondary or exploratory.

In addition, exploratory correlation analyses will be conducted to examine relationships both among the different outcomes from the tools and between these outcomes and summary measures of the biometrical parameters recorded by the wearable devices. Depending on the distribution of the variables, Pearson or Spearman correlation coefficients will be used. For these analyses, the correlation coefficient r and its 95% CI will be reported as the primary measure of effect size. To explore relationships between categorical variables, the chi-square test will be applied. When expected cell frequencies are low, the Fisher exact test will be used instead.

Furthermore, given the longitudinal nature of the study, data from wearable devices and scores from the weekly questionnaire will be summarized at the weekly level. For wearable data, daily recordings will be checked for validity and aggregated as weekly averages. Based on previous studies [64,65], a week will be considered valid if it contains at least 4 days with usable recordings. Trends in weekly measurements of sleep, heart rate, and step count, as well as weekly questionnaire scores, will be analyzed using repeated-measures ANOVA or the nonparametric Friedman test, depending on data distribution, including only participants who meet prespecified minimum completeness criteria for the weekly data.

To handle incomplete weekly or wearable data systematically and reduce the risk of biased estimates, the amount and pattern of missing wearable and weekly questionnaire data will be described, and all available valid weeks will be included in the analyses under a missing-at-random assumption. In addition, sensitivity analyses will be performed to assess the robustness of the longitudinal and correlational findings to different assumptions about missing data.

Regarding the qualitative analysis of the final interview, the instructions of the thematic analysis by Braun and Clarke [66] will be followed, who defined 6 phases: (1) familiarizing with the data; (2) generating initial codes; (3) searching for themes; (4) reviewing themes; (5) defining and naming themes; and (6) producing the report. The analysis will be carried out independently by 3 researchers using the previously described inductive approach, and MAXQDA 24 software [67] will be used for the triangulation of the information.

## Results

**Figure 2.**
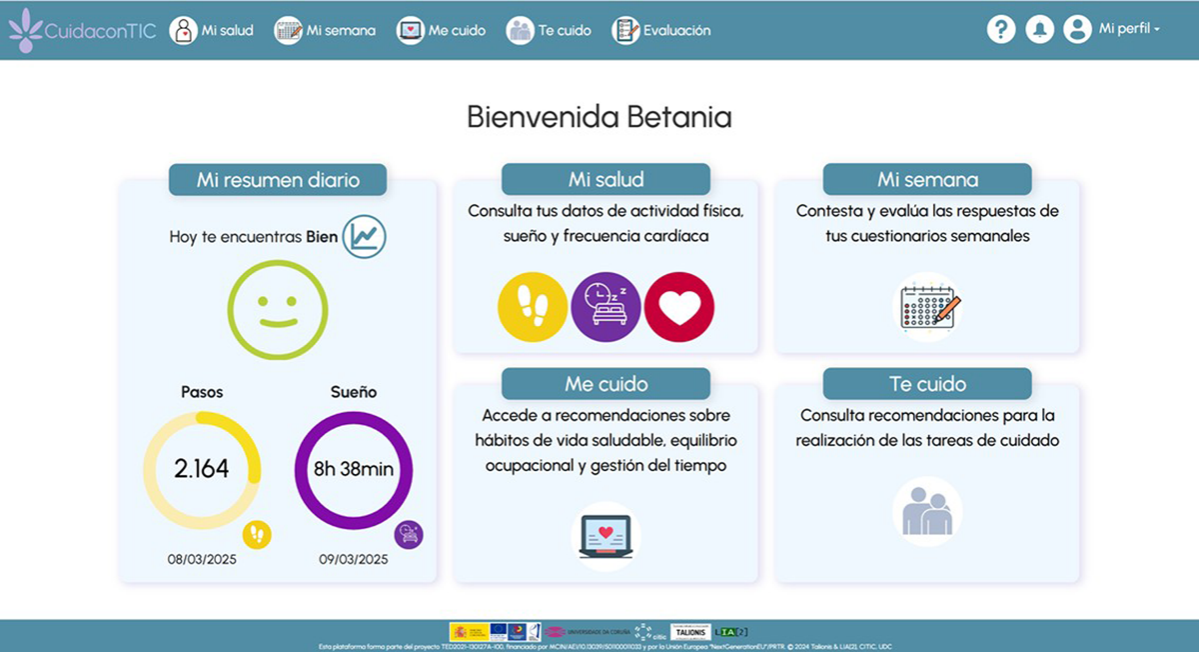
Screenshot of the *CuidaconTIC* platform.

The project was funded in December 2022, and enrollment was completed in March 2025. As of October 2025, this study has completed the recruitment of participants (N=62). The improvements resulting from the iterative co-design process with caregivers have now been implemented on the platform. Figure 2 shows the main menu of the *CuidaconTIC* platform in Spanish with the main sections: My daily summary, My health, My week, I take care of myself, and I take care of you.

The dataset is now being prepared for publication. Data analysis (quantitative and qualitative) will begin next, and the first results are expected to be submitted for publication in June 2026.

## Discussion

### Anticipated Findings

This study aims to develop and evaluate an innovative technological solution aimed at enhancing the QoL, impact of care, occupational balance, self-management of health, and empowerment of informal caregivers of older people and/or people with disabilities.

Technology, empowerment, and the PCC framework can help to improve the caregiver’s health status, QoL, and self-management of their own health. These benefits could extend to the care recipients. Hence, caring for the caregiver has a double effect and greater impact on the social and health systems [68]. The PCC approach is a core element of this research, widely studied in health sciences [21,69,70]. The design and development of the *CuidaconTIC* platform will study the eight PCC dimensions: (1) respect for individuals’ values, preferences, and expressed needs; (2) provision of information and education; (3) access to care; (4) emotional support; (5) involvement of family and friends; (6) continuity and safe transitions; (7) physical comfort, and (8) care coordination.

Person-centered technology has significant potential to support informal caregivers. However, most existing technological solutions primarily focus on care recipients, aiming to promote their autonomy, improve their QoL, and consequently, reduce the burden on caregivers [33,71]. Although there are initiatives targeted at caregivers, they tend to be designed for specific groups, such as caregivers of older adults with dementia, or they primarily provide training and informational services without addressing the broader needs of informal care.

Following a comprehensive analysis of health care and social challenges faced by informal caregivers, this study will propose alternative solutions using person-centered technology. This approach will help advance the field of care and technology as a tool to support caregivers in their daily life.

If the initial hypothesis is confirmed, the study will move toward exploring a low-cost intervention with minimal time requirements, oriented in particular for people with limited availability.

To our knowledge, this is one of the first studies that places informal caregivers at the center of technological development, involving them in an iterative process to create a tool that not only improves care but also directly supports their own QoL, the impact of care, occupational balance, self-management of health, and empowerment.

In summary, this study has the potential to improve caregivers’ perception of their own QoL and health status, promote healthy lifestyle habits, and expand knowledge about care and needs from a preventive perspective.

### Conclusions

This trial focuses on studying the social challenge of the QoL of the caregiver population, mostly women, and on reducing the digital gap and promoting inclusion, through a service that places people and care at the center.

The results of this study will provide an in-depth understanding of the phenomenon of informal care. In addition, it will explore from the perspective of the caregivers themselves what aspects can be interesting to support their daily life through a technological solution focused on the care for caregivers.

## Supplementary material

10.2196/86602 Multimedia Appendix 1 Summary of the trial, based on the WHO Trial Registration Data Set. WHO: World Health Organization.

10.2196/86602 Multimedia Appendix 2 Semistructured interview guide.

## Abbreviations

CarerQoL: care-related quality of life
CONSORT-EHEALTH: Consolidated Standards of Reporting Trials of Electronic and Mobile Health Applications and Online Telehealth
PCC: person-centered care
QoL: quality of life
SPIRIT: Standard Protocol Items: Recommendations for Interventional Trials
VAS: visual analog scale
