# Supplementary material for: Enhancing Caregivers’ Quality of Life Through a Web-Based Person-Centered Solution (TechQoL4Carers): Protocol for a Mixed Methods Pilot Trial
Source: JMIR Res Protoc. 2026 Feb 5;15:e86602. doi: 10.2196/86602 (PMC12875564; doi:10.2196/86602)
Supplement: Multimedia Appendix 1 [file resprot-v15-e86602-s001.docx]

# Summary of the Trial, Based on the WHO Trial Registration Data Set

| **Data category** | **Information** |
| --- | --- |
| Primary registry and trial identifying number | ClinicalTrials.gov. ID: NCT06226285 |
| Date of registration in primary registry | 24 January, 2024 |
| Secondary identifying numbers | TED2021-130127A-I00; 2023_019 |
| Source(s) of monetary or material support | Ministry of Science and Innovation, Spanish Government |
| Primary sponsor | Universidade da Coruña (University of A Coruña) |
| Secondary sponsor(s) | Center on Information and Communication Technologies (CITIC) |
| Contact for public queries | [proxectocuidacontic@udc.es](mailto:proxectocuidacontic@udc.es)  0034981167000 ext 5870  Address: Universidade da Coruña, Talionis Research Group, Campus de Oza, As Xubias 15071, A Coruña, Spain |
| Contact for scientific queries | Betania Groba, Principal Investigator, [b.groba@udc.es](mailto:b.groba@udc.es)  Laura Nieto-Riveiro, Principal Investigator, [laura.nieto@udc.es](mailto:laura.nieto@udc.es);  Universidade da Coruña, CITIC, Talionis Research Group  0034981167000 ext 5870  Address: Universidade da Coruña, Talionis Research Group, Campus de Oza, As Xubias 15071, A Coruña, Spain |
| Public title | Quality of Life for Caregivers through a Person-Centered Technological Solution (TechQoL4Carers) |
| Scientific title | Quality of Life for Caregivers Through a Person-Centered Technological Solution |
| Countries of recruitment | Spain |
| Health condition(s) or problem(s) studied | Informal caregivers, Quality of Life, technology, activities of daily living |
| Intervention(s) | The technological platform of the TechQoL4Carers project, under the name *CuidaconTIC* |
| Key inclusion and exclusion criteria | Ages eligible for study: ≥ 18 years; Sexes eligible for study: all; Accepts healthy volunteers: yes |
|  | Inclusion criteria: perform the role of informal caregiver, being the main caregiver, live with the person receiving the care, regular smartphone use; compromise for wearing a wearable wristband |
|  | Exclusion criteria: < one year in the informal caregiver role, care for more than one person in a situation of dependency, have modified legal capacity |
| Study type | Interventional |
|  | Allocation: N/A |
|  | Primary purpose: Supportive care |
|  | Phase: N/A |
| Date of first enrolment | May 2024 |
| Target sample size | 54 |
| Recruitment status | Recruiting |
| Primary outcome(s) | Health-related Quality of Life (QoL), overall health, care-related QoL, well-being  Time frame: baseline, mid-term, and post-intervention |
| Key secondary outcomes | Caregiver burden, caregiver strain, occupational balance, empowerment. Time frame: baseline, mid-term, and post-intervention  Physical activity and sleep. Time frame: 3 months wearing the Xiaomi Smart Band  Daily functioning: Time frame: once a week during the intervention  Satisfaction with the project, usability, and meaning attributed to participation in the project. Timeframe: post-intervention |
