# Supplementary material for: Enhancing Caregivers’ Quality of Life Through a Web-Based Person-Centered Solution (TechQoL4Carers): Protocol for a Mixed Methods Pilot Trial
Source: JMIR Res Protoc. 2026 Feb 5;15:e86602. doi: 10.2196/86602 (PMC12875564; doi:10.2196/86602)
Supplement: Multimedia Appendix 2 [file resprot-v15-e86602-s002.docx]

# Appendix B. Semi-Structured Interview Guide

**General aspects**

Could you describe your overall experience with this project and with the *CuidaconTIC* platform?

What do you consider to be the strengths of this platform? Why?

And what do you think are the areas for improvement or what would you change? Why?

Could you explain what the use of this platform has brought to your day-to-day life?

**Occupational balance**

How would you describe your occupational balance in your daily life?

After these months of use, how do you think the platform has helped you maintain the balance between your daily activities?

**Impact of care**

How would you describe the caring tasks you perform?

And what has the use of *CuidaconTIC* brought to the management and execution of these caregiving tasks?

**Empowerment and self-management of health**

How capable do you now feel of taking care of yourself?

And how capable do you now feel of taking care of your family member?

In general, how do you think the use of this platform can help you make better decisions?

**Quality of life**

What level of satisfaction and happiness have you felt in the last three months?

Could you tell me what impact the participation in this project and the use of *CuidaconTIC* have had on your quality of life?

**Closing**

After participating in this experience, what do you think technology can offer to caregivers?
